# Hemoglobin Trajectory Phenotypes and Neurological Outcomes in a Neurosurgical ICU Cohort

**DOI:** 10.3390/jcm15135254

**Published:** 2026-07-05

**Authors:** Yoonhee Hong, Jeong-Am Ryu

**Affiliations:** 1 Department of Critical Care Medicine, Samsung Medical Center, School of Medicine, Sungkyunkwan University, 81 Irwon-ro, Gangnam-gu, Seoul 06351, Republic of Korea; yoonhee.hong@samsung.com; 2 Department of Neurosurgery, Samsung Medical Center, School of Medicine, Sungkyunkwan University, 81 Irwon-ro, Gangnam-gu, Seoul 06351, Republic of Korea

**Keywords:** hemoglobins, critical care, brain injuries, blood transfusion, phenotype

## Abstract

**Background/Objectives:** Current hemoglobin management in neurocritical care relies on static transfusion thresholds, which fail to capture the dynamic nature of hemoglobin changes during ICU care. We aimed to determine whether distinct longitudinal hemoglobin trajectory phenotypes exist among neurosurgical ICU patients and whether specific trajectory patterns independently predict unfavorable neurological outcomes. **Methods:** In this retrospective observational cohort study, we analyzed 8517 patients admitted to the neurosurgical ICU of a tertiary academic medical center between January 2015 and December 2024. A feature-based Gaussian mixture model was applied to trajectory-derived hemoglobin features over the first 14 ICU days to identify distinct hemoglobin trajectory phenotypes. The association between trajectory class and neurological outcomes was evaluated using propensity score matching. **Results:** Six distinct hemoglobin trajectory phenotypes were identified. The “Rapid Dropper” phenotype (Class 2; *n* = 351, 4.1%), characterized by the steepest decline velocity and highest variability, showed dramatically worse outcomes: 36.5% unfavorable neurological outcome (Glasgow Outcome Scale 1–3) versus 3.1% in all other classes combined (odds ratio [OR], 18.10; 95% confidence interval [CI], 14.08–23.27). This association persisted after propensity score matching (OR, 2.40; 95% CI, 1.77–3.26; *p* < 0.001). Hemorrhagic diagnoses were disproportionately concentrated in this high-risk phenotype. A combined prediction model incorporating trajectory-derived features within 72 h achieved an area under the receiver operating characteristic curve of 0.850 (95% CI, 0.829–0.870). This value reflects retrospective full-trajectory phenotyping; in a 72 h landmark analysis, early-feature prediction achieved an AUC of approximately 0.72. **Conclusions:** Hemoglobin trajectory phenotyping identified a high-risk “Rapid Dropper” subgroup that was significantly associated with worse short-term neurological outcomes. The rate of hemoglobin decline, rather than any single threshold, was associated with prognostic separation; prospective and external validation is required before these associations can inform transfusion strategy.

## 1. Introduction

Anemia is one of the most frequently encountered complications in neurocritically ill patients, with reported prevalence rates ranging from 20% to 50% during intensive care unit (ICU) stays [1,2]. In the injured brain, where cerebrovascular reserve is often compromised, hemoglobin serves as the principal determinant of arterial oxygen content and, consequently, cerebral oxygen delivery [2,3]. Experimental and clinical studies have consistently demonstrated that progressive reductions in hemoglobin concentration impair brain tissue oxygenation, particularly when levels fall below 9 g/dL, at which point compensatory cerebral vasodilation becomes insufficient to maintain adequate oxygen supply [3,4]. Given that secondary ischemic injury remains a major contributor to neurological deterioration in patients with subarachnoid hemorrhage (SAH), intracerebral hemorrhage (ICH), and traumatic brain injury (TBI), optimal hemoglobin management is of paramount importance in the neurocritical care setting [2,5].

Current research on hemoglobin management in neurocritical care has predominantly focused on defining the optimal transfusion threshold, debating whether a restrictive (hemoglobin ≤ 7–8 g/dL) or liberal (hemoglobin ≤ 9–10 g/dL) strategy yields superior outcomes [6,7,8,9]. Recent landmark trials, including the HEMOTION trial in traumatic brain injury [6] and the SAH-RCT trial in aneurysmal SAH [7], along with the TRAIN multicenter trial [8], have produced mixed results. Meta-analyses suggest that liberal transfusion strategies may confer modest neurological benefits in certain acute brain injury populations [9,10], yet the debate remains unresolved and consensus guidelines continue to evolve. Notably, the Cochrane systematic review on transfusion thresholds concluded that neurocritically ill patients may represent a unique population requiring differentiated approaches compared to general ICU patients [10]. However, this binary, threshold-based paradigm inherently oversimplifies the complex and dynamic nature of hemoglobin physiology in the injured brain.

A critical limitation of the threshold-based framework is its static nature: it captures hemoglobin at a single time point rather than characterizing its longitudinal behavior over the course of ICU care. Emerging evidence from other critical care populations suggests that the trajectory of biomarker changes—including rate of decline, variability, and overall trend—may carry greater prognostic significance than any single measurement [11,12]. Physiologically, this concept is particularly compelling in the neurocritical care setting. The injured brain exhibits impaired cerebral autoregulation, with a narrowed cerebrovascular reserve that limits compensatory vasodilation in response to declining hemoglobin [2,13]. Consequently, a rapid decline in hemoglobin may produce cumulative and sustained reductions in cerebral oxygen delivery that exceed the brain’s capacity for compensation, even if the absolute hemoglobin nadir remains above conventional transfusion thresholds [3,14]. Furthermore, hemoglobin variability—reflecting fluctuations in oxygen-carrying capacity—may impose repetitive perfusion challenges on vulnerable brain tissue, compounding the risk of secondary ischemic injury [13,15].

Finite mixture models, including the Gaussian mixture model, provide a robust statistical framework for identifying latent subgroups of individuals who follow distinct longitudinal patterns of change over time [16,17,18]. Originally developed in the social and behavioral sciences, such trajectory-clustering approaches have been increasingly applied in clinical research to uncover hidden phenotypic heterogeneity within ostensibly homogeneous patient populations [16,17]. Recent studies have demonstrated the utility of trajectory-based approaches for various ICU biomarkers, including lactate, albumin, and organ dysfunction scores, revealing that longitudinal patterns consistently outperform single time-point measurements in predicting clinical outcomes [11,18,19]. In the field of hemoglobin research, trajectory modeling has been applied to cardiac surgery populations, identifying distinct hemoglobin evolution patterns associated with acute kidney injury [12]. However, the application of hemoglobin trajectory phenotyping to neurocritical care populations—where the relationship between hemoglobin dynamics and cerebral perfusion carries uniquely critical implications—has not been explored.

We hypothesized that distinct hemoglobin trajectory phenotypes exist among neurosurgical ICU patients, and that specific trajectory patterns characterized by rapid hemoglobin decline and high variability are significantly associated with unfavorable neurological outcomes, beyond what is captured by initial hemoglobin level or absolute nadir alone. To test this hypothesis, we applied a feature-based Gaussian mixture model to a large cohort of patients admitted to a neurosurgical ICU and systematically evaluated the association between trajectory phenotypes and neurological outcomes using propensity score–matched analyses. We further examined the predictive performance of trajectory-derived features, including slope, variability, and delta hemoglobin, as clinically accessible biomarkers for early risk stratification. By shifting the paradigm from static threshold-based to dynamic trajectory-based hemoglobin assessment, this study aims to provide novel insights into hemoglobin management and prognostication in neurocritical care [20].

## 2. Materials and Methods

### 2.1. Study Design and Population

This was a retrospective, single-center, observational study conducted on patients admitted to the neurosurgical ICU of Samsung Medical Center, a tertiary referral hospital in Seoul, Korea, between January 2015 and December 2024. This study was reviewed by the Institutional Review Board (IRB) of Samsung Medical Center, which granted an exemption from review (File No. SMC 2026-01-145) on 29 January 2026. As this was a retrospective study based on existing medical records, the IRB waived the requirement for informed consent. The study was conducted in accordance with the Declaration of Helsinki (1975, revised in 2013). We included patients who were hospitalized in the neurosurgical ICU for the management of neurocritical illness or postoperative care following neurosurgery. Neurocritically ill patients were defined as those admitted for brain tumor surgery, SAH, ICH, TBI, or unruptured cerebral aneurysm surgery. Patients were excluded if they had fewer than two hemoglobin measurements during their ICU stay, or incomplete outcome data at hospital discharge.

### 2.2. Data Collection

Clinical data were retrospectively extracted from the hospital’s Clinical Data Warehouse. Our center constructed the “Clinical Data Warehouse Darwin-C” designed for investigators to search and retrieve de-identified medical records from the electronic archives. It contains data pertaining to more than 4 million patients. The clinical and laboratory data were extracted from the Clinical Data Warehouse Darwin-C after finalizing the patient list in this study [1]. Baseline characteristics included age, sex, primary neurosurgical diagnosis, and comorbidities (hypertension, diabetes mellitus, malignancy, cardiovascular disease, and cerebrovascular disease). The Glasgow Coma Scale (GCS) score at ICU admission was recorded as a measure of neurological severity. ICU interventions including mechanical ventilation, continuous renal replacement therapy (CRRT), and intracranial pressure (ICP) monitoring were documented. All hemoglobin measurements obtained during the first 14 days of ICU admission were collected. Red blood cell (RBC) transfusion data, including number of units and transfusion events, were also recorded.

This cohort comprised predominantly elective and postoperative neurosurgical patients (brain tumor 48.9%, other neurosurgical conditions 26.1%, unruptured aneurysm 17.2%, and moyamoya disease 3.4%), whereas classic neurocritical diagnoses (subarachnoid hemorrhage 1.7%, traumatic brain injury 1.6%, and intracerebral hemorrhage 1.2%) together accounted for only 4.4%. We therefore frame this study as a neurosurgical ICU cohort that shares a common risk of postoperative bleeding and hemoglobin variation, rather than as representative of neurocritical illness broadly.

### 2.3. Hemoglobin Trajectory Phenotyping

To identify distinct longitudinal hemoglobin phenotypes over the first 14 ICU days, we used a feature-based clustering approach. For each patient, serial hemoglobin measurements were aggregated into patient-level daily means (days 0–13); patients were required to have at least three observed days of values (median 4, IQR 3–6), and missing intermediate days within the observed range were filled by linear interpolation between adjacent observed days, which accounted for approximately 41% of the day-level values used for feature computation. From this daily series we derived three features summarizing the trajectory: the intercept (baseline level) and slope (overall rate of change, g/dL/day) from an ordinary least-squares regression of hemoglobin on time, and the within-patient standard deviation of hemoglobin (variability). The three standardized features were clustered using a Gaussian mixture model (full covariance; scikit-learn, Python 3.10 (Python Software Foundation, Wilmington, DE, USA); a fixed random seed of 42 and 10 random initializations) [21,22]. The number of phenotypes was selected over a pre-specified range of two to six components using the Bayesian Information Criterion [23], supported by the average posterior probability of assignment (all ≥ 0.67) and clinical interpretability; a six-phenotype solution was selected, and each patient was assigned to the phenotype with the highest posterior probability. Candidate model-fit statistics and posterior probabilities are reported in the Supplementary Materials (Tables S1 and S2). Delta hemoglobin (ΔHb; first minus minimum hemoglobin) and mean hemoglobin level were retained, with slope and variability, as continuous trajectory-derived features for subsequent analyses.

### 2.4. Outcomes

The primary outcome was unfavorable neurological outcome at hospital discharge, defined as Glasgow Outcome Scale (GOS) score of 1–3 (death, persistent vegetative state, or severe disability). Secondary outcomes included in-hospital mortality, 28-day mortality, and ICU mortality. All outcomes were assessed by the treating clinical team and documented in the electronic medical record. Secondary clinical measures included intensive care unit and hospital length of stay; in the subset of patients with an available perioperative baseline hemoglobin, the magnitude and timing of the perioperative hemoglobin decline to the 7-day nadir were also examined.

### 2.5. Statistical Analysis

Continuous variables are presented as mean ± standard deviation and compared using one-way analysis of variance (ANOVA) or the Kruskal–Wallis test, as appropriate. Categorical variables are expressed as counts (percentages) and compared using the chi-squared test or Fisher’s exact test. Unadjusted odds ratios (ORs) with 95% confidence intervals (CIs) were calculated using Fisher’s exact test for each outcome comparing Class 2 versus all other classes. To account for baseline differences between Class 2 (Rapid Dropper) and other trajectory classes, propensity score matching (PSM) was performed using a 1:2 nearest-neighbor matching algorithm with a caliper of 0.2 standard deviations of the logit of the propensity score [24]. The propensity score was estimated using logistic regression with the following covariates: age, sex, initial hemoglobin, GCS score, hypertension, diabetes mellitus, malignancy, and primary diagnosis SAH, ICH, TBI, brain tumor, and unruptured aneurysm. Covariate balance was assessed using standardized mean differences (SMDs), with SMD < 0.1 indicating adequate balance. After matching, ORs with 95% CIs were recalculated for all outcomes [24,25]. Subgroup analyses were performed stratified by primary neurosurgical diagnosis to evaluate the consistency of the association between Class 2 membership and unfavorable neurological outcome across diagnostic categories. Diagnosis-specific ORs with 95% CIs were computed using Fisher’s exact test. The predictive performance of hemoglobin trajectory features for unfavorable neurological outcome was assessed using receiver operating characteristic (ROC) analysis. Individual hemoglobin features (ΔHb, slope, and variability) were evaluated as single predictors, and combined models incorporating three features (ΔHb, slope, and variability) were constructed using logistic regression. Discrimination was quantified using the area under the ROC curve (AUC), and internal validation was performed using 5-fold stratified cross-validation. Sensitivity, specificity, positive predictive value (PPV), and negative predictive value (NPV) of Class 2 membership as a binary predictor of each outcome were also reported. All statistical analyses were performed using Python 3.10 (Python Software Foundation) with the scikit-learn, SciPy, and pandas libraries. A two-sided *p*-value < 0.05 was considered statistically significant.

### 2.6. Sensitivity and Early-Prediction Analyses

Several pre-specified sensitivity analyses were performed to address potential confounding and bias. To address residual confounding, in addition to the pre-specified 12-covariate propensity-score model, we performed a sensitivity analysis further adjusting for illness severity (APACHE II) and treatment intensity (mechanical ventilation, intracranial pressure monitoring, and continuous renal replacement therapy). Because hemoglobin sampling frequency differed across phenotypes, we additionally adjusted for the number of hemoglobin measurements. Because trajectory classification was based on raw hemoglobin values only and transfusion was not used as a classifying variable, transfusion effects were assessed using (i) a never-transfused subgroup and (ii) a transfusion-adjusted model. To separate retrospective 14-day phenotyping from genuine early prediction, we performed a landmark analysis restricted to hemoglobin data from the first 72 h, using four early features (first hemoglobin, minimum hemoglobin, ΔHb, and slope) to predict unfavorable outcome (*n* = 8495; 99.7% of the cohort). Finally, to assess the influence of interpolation, we repeated phenotyping and the prognostic analyses using observed daily means only, without interpolation.

## 3. Results

### 3.1. Study Population and Hemoglobin Trajectory Phenotypes

Of 13,663 patients admitted to the neurosurgical ICU between January 2015 and December 2024, 5146 were excluded due to age < 18 years (*n* = 423), do-not-resuscitate orders (*n* = 689), transfer from other hospitals with insufficient data (*n* = 1300), incomplete medical records (*n* = 872), incomplete hemoglobin data (*n* = 774), missing Glasgow Outcome Scale data (*n* = 821), and non-neurocritical diagnosis (*n* = 267), yielding a final cohort of 8517 patients (Supplemental Figure S1). The mean age was 55.2 ± 14.5 years, and 3573 patients (41.9%) were male. The most common primary diagnoses were brain tumor (4165, 48.9%), other neurosurgical conditions (2222, 26.1%), and unruptured aneurysm (1461, 17.2%), followed by moyamoya disease (290, 3.4%), SAH (141, 1.7%), TBI (138, 1.6%), and ICH (100, 1.2%). The mean initial hemoglobin was 11.9 ± 1.7 g/dL, and the mean GCS score was 14.4 ± 1.8. Comorbidities included malignancy (5260, 61.8%), cerebrovascular disease (2721, 31.9%), hypertension (844, 9.9%), and diabetes mellitus (509, 6.0%). Mechanical ventilation was used in 1191 patients (14.0%), ICP monitoring in 640 (7.5%), and CRRT in 22 (0.3%). Overall, 1475 patients (17.3%) received RBC transfusions during their ICU stay (Table 1).

**Figure 1:**
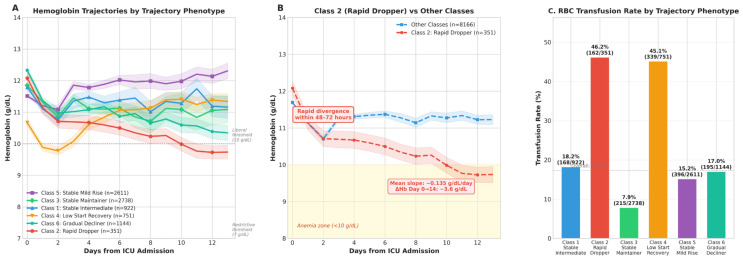
Hemoglobin Trajectories by Trajectory Phenotype. (**A**) Mean hemoglobin trajectories over the first 14 days of ICU admission for each of the six trajectory classes identified by Gaussian mixture modeling. Shaded areas represent 95% confidence intervals. Dashed lines indicate the liberal (10 g/dL) and restrictive (7 g/dL) transfusion thresholds. Class 5 (Stable Mild Rise; *n* = 2611, 30.7%) and Class 3 (Stable Maintainer; *n* = 2738, 32.1%) were the two largest groups with stable trajectories. Class 2 (Rapid Dropper; *n* = 351, 4.1%) exhibited the steepest hemoglobin decline. (**B**) Comparison of Class 2 versus all other classes combined (*n* = 8166), demonstrating rapid hemoglobin divergence within 48–72 h and entry into the anemia zone (<10 g/dL) by day 10. Mean slope of Class 2 was −0.135 g/dL/day with a total hemoglobin drop of approximately 3.6 g/dL over 14 days. (**C**) RBC transfusion rates by trajectory class. Class 2 (46.2%, 162/351) and Class 4 (45.1%, 339/751) showed the highest transfusion rates, compared to an overall rate of 17.3% (1475/8517).

Gaussian mixture modeling of trajectory-derived features identified six distinct hemoglobin trajectory phenotypes (Figure 1A). Class 3 (Stable Maintainer; *n* = 2738, 32.1%) and Class 5 (Stable Mild Rise; *n* = 2611, 30.7%) were the two largest groups, together comprising 62.8% of the cohort. Class 1 (Stable Intermediate; *n* = 922, 10.8%) and Class 6 (Gradual Decliner; *n* = 1144, 13.4%) represented moderate-sized groups with relatively stable or gradually declining hemoglobin trajectories. Class 4 (Low Start Recovery; *n* = 751, 8.8%) demonstrated an initial hemoglobin dip followed by recovery, with the lowest initial hemoglobin (11.2 ± 1.8 g/dL) and a positive slope (+0.132 g/dL/day). Class 2 (Rapid Dropper; *n* = 351, 4.1%) was the smallest group and exhibited the most clinically concerning trajectory, with the steepest negative slope (−0.135 ± 0.115 g/dL/day), the largest ΔHb (3.62 ± 1.71 g/dL), and the highest hemoglobin variability (1.09 ± 0.34). Trajectory class characteristics are summarized in Table 1.

### 3.2. Class 2 (Rapid Dropper) Phenotype

**Figure 2:**
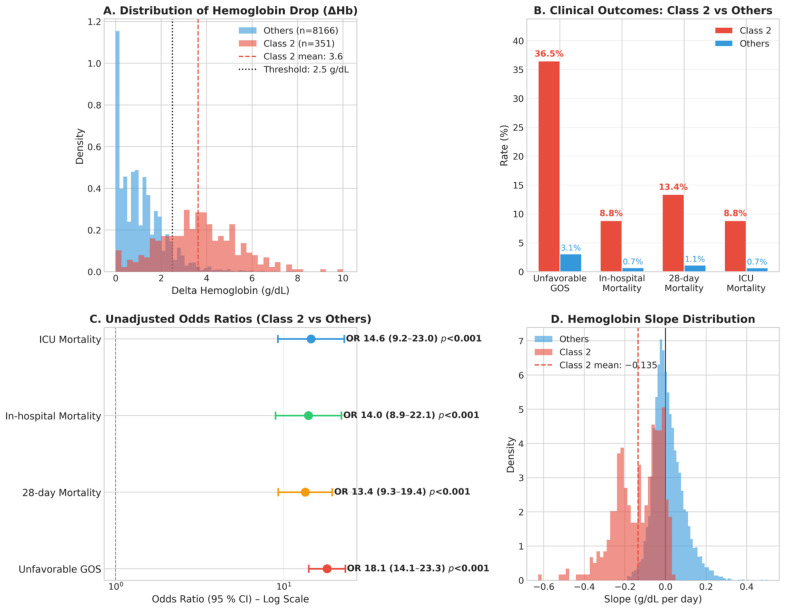
Detailed Analysis of Class 2 (Rapid Dropper) Phenotype. (**A**) Distribution of hemoglobin drop (ΔHb, defined as first Hb minus minimum Hb) comparing Class 2 (*n* = 351, red) versus other classes (*n* = 8166, blue). Class 2 patients had a mean ΔHb of 3.6 g/dL compared to 1.1 g/dL in others. The dotted line indicates the ΔHb threshold of 2.5 g/dL. (**B**) Comparison of clinical outcome rates between Class 2 and other classes. Unfavorable neurological outcome (36.5% vs. 3.1%), in-hospital mortality (8.8% vs. 0.7%), 28-day mortality (13.4% vs. 1.1%), and ICU mortality (8.8% vs. 0.7%) were all significantly higher in Class 2. (**C**) Forest plot showing unadjusted odds ratios (log scale) for Class 2 versus other classes across all outcome measures: unfavorable GOS (OR 18.1, 95% CI 14.1–23.3), ICU mortality (OR 14.6, 95% CI 9.2–23.0), in-hospital mortality (OR 14.0, 95% CI 8.9–22.1), and 28-day mortality (OR 13.4, 95% CI 9.3–19.4); all *p* < 0.001. (**D**) Distribution of hemoglobin slope (g/dL per day) comparing Class 2 versus others. Class 2 showed a uniquely steep negative slope (mean −0.135 g/dL/day) compared to near-zero or positive slopes in other classes.

Comparison of the Class 2 trajectory with all other classes combined revealed rapid divergence of hemoglobin levels within 48–72 h of ICU admission (Figure 1B). By day 10, mean hemoglobin in Class 2 dropped below 10.0 g/dL, entering the anemia zone, while the aggregate trajectory of other classes remained above 11.0 g/dL. Class 2 patients had the highest rates of RBC transfusion (46.2%, 162/351) and mechanical ventilation (58.4%), compared to the overall cohort rates of 17.3% and 14.0%, respectively (Figure 1C, Table 1). Class 4 (Low Start Recovery) also demonstrated high transfusion rates (45.1%, 339/751), likely to reflect the initial low hemoglobin values that prompted both transfusion and the subsequent recovery trajectory. The distribution of ΔHb showed clear separation between Class 2 and other classes, with a mean ΔHb of 3.62 g/dL versus 1.13 g/dL (Figure 2A). Similarly, the hemoglobin slope distribution confirmed Class 2’s uniquely steep decline (mean −0.135 g/dL/day) compared to near-zero or positive slopes in other classes (Figure 2D).

### 3.3. Clinical and Neurological Outcomes

**Figure 3:**
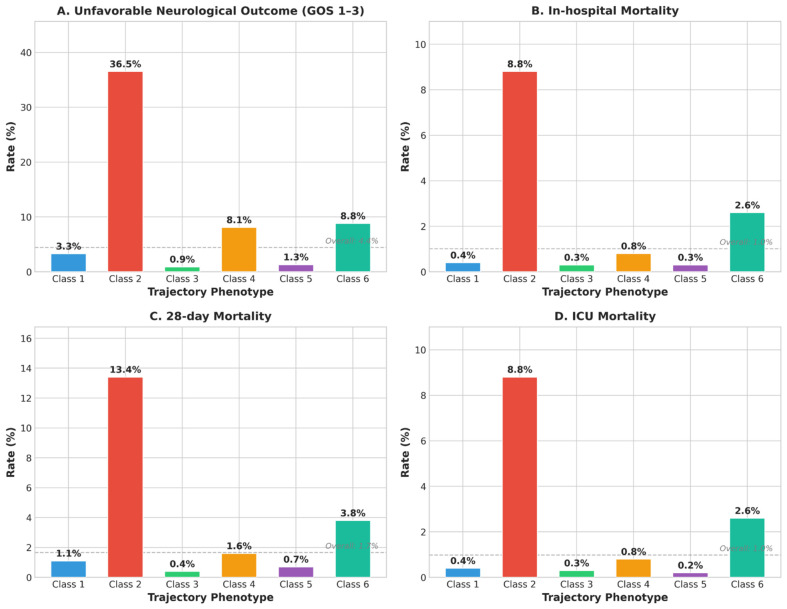
Clinical Outcomes by Hemoglobin Trajectory Phenotype. (**A**) Unfavorable neurological outcome (GOS 1–3) rates by trajectory class. Class 2 (Rapid Dropper) exhibited a dramatically elevated rate (36.5%) compared to all other classes (range: 0.9–8.8%). The overall rate was 4.4% (dashed line). (**B**) In-hospital mortality rates by trajectory class. Class 2 had the highest rate (8.8%) followed by Class 6 (Gradual Decliner, 2.6%). (**C**) 28-day mortality rates by trajectory class. Class 2 demonstrated the highest rate (13.4%), markedly exceeding the overall rate of 1.6%. (**D**) ICU mortality rates by trajectory class. Class 2 showed the highest ICU mortality (8.8%), compared to rates of 0.2–2.6% in other classes.

The overall rate of unfavorable neurological outcome (GOS 1–3) was 4.4% (379/8517). Clinical outcomes differed dramatically across trajectory classes (Figure 3). Class 2 patients exhibited the highest rates of all adverse outcomes: unfavorable neurological outcome (36.5%, 128/351), 28-day mortality (13.4%, 47/351), in-hospital mortality (8.8%, 31/351), and ICU mortality (8.8%, 31/351). The higher 28-day mortality compared to in-hospital mortality in Class 2 reflects 16 additional deaths occurring after hospital discharge but within the 28-day observation window, likely among patients discharged with severe neurological impairment. In contrast, Class 3 (Stable Maintainer) and Class 5 (Stable Mild Rise) had the lowest outcome rates, with unfavorable neurological outcome rates of 0.9% and 1.3%, respectively.

In unadjusted analysis, Class 2 membership was associated with markedly elevated odds of all adverse outcomes compared to all other classes combined (Figure 2C): unfavorable neurological outcome (OR 18.10, 95% CI 14.08–23.27; *p* < 0.001), 28-day mortality (OR 13.42, 95% CI 9.28–19.41; *p* < 0.001), in-hospital mortality (OR 14.03, 95% CI 8.92–22.06; *p* < 0.001), and ICU mortality (OR 14.55, 95% CI 9.23–22.95; *p* < 0.001). Class 6 (Gradual Decliner) also demonstrated elevated outcome rates (unfavorable GOS 8.8%, 28-day mortality 3.8%), suggesting that any declining hemoglobin trajectory, regardless of the rate of decline, may confer increased risk.

### 3.4. Propensity Score Matched Analysis

Propensity score matching yielded 329 Class 2 patients matched with 643 controls (Supplemental Figure S3A). Adequate covariate balance was achieved after matching, with all SMDs reduced to within the ±0.1 threshold (Supplemental Figure S3B). After PSM, the odds ratios were substantially attenuated but remained significant for the primary outcome and 28-day mortality (Supplemental Figure S3C,D): unfavorable neurological outcome (OR 2.40, 95% CI 1.77–3.26; *p* < 0.001) and 28-day mortality (OR 2.09, 95% CI 1.33–3.28; *p* = 0.002). In-hospital mortality (OR 1.59, 95% CI 0.93–2.70; *p* = 0.090) and ICU mortality (OR 1.64, 95% CI 0.96–2.80; *p* = 0.085) showed trends toward significance but did not reach the conventional threshold after matching, suggesting that the extreme unadjusted ORs were partly confounded by baseline severity differences.

In sensitivity analyses, the association between the Rapid Dropper phenotype and unfavorable outcome was robust to additional adjustment. After further adjustment for APACHE II and treatment intensity, Class 2 remained significantly associated with unfavorable GOS (OR 4.98; 95% CI, 3.31–7.50; *p* < 0.001) and 28-day mortality (OR 2.49; 95% CI, 1.52–4.08; *p* < 0.001), whereas ICU mortality (OR 1.57; 95% CI, 0.84–2.94) and in-hospital mortality (OR 1.52; 95% CI, 0.81–2.85) were not significant (Table S3). Among never-transfused patients (*n* = 7042; Class 2 *n* = 189), the phenotype remained associated with unfavorable GOS (adjusted OR 6.56; 95% CI, 3.57–12.04; *p* < 0.001) and 28-day mortality (OR 3.44; 95% CI, 1.53–7.72; *p* = 0.003), and associations persisted when transfusion was added to the model (unfavorable GOS OR 6.67 [95% CI, 4.50–9.90]; 28-day mortality OR 2.61 [95% CI, 1.59–4.30]). Class 2 also had a higher hemoglobin sampling frequency (median ≈ 13 vs. 3–7 measurements); after adjustment for the number of hemoglobin measurements, associations persisted (unfavorable GOS OR 2.71 [95% CI, 1.74–4.20]; 28-day mortality OR 3.08 [95% CI, 1.79–5.31]; both *p* < 0.001).

The Rapid Dropper phenotype was also associated with markedly longer stays. Class 2 had longer ICU (median 3.1 [IQR 0.9–11.6] days) and hospital (27.8 [15.2–51.1] days) length of stay than other phenotypes (overall 1.0 and 8.9 days; Kruskal–Wallis *p* < 0.001; Class 2 vs. others *p* < 0.001; Table S4). Among patients with an available perioperative baseline hemoglobin (*n* = 980, 11.5%), hemoglobin fell by a median of 2.3 g/dL (18%) to the 7-day nadir; the decline was greatest in the Rapid Dropper phenotype (3.6 g/dL, 26%), which reached its nadir latest (median 3.9 vs. 0.7–2.6 days), indicating a progressive rather than single-event decline (*p* < 0.001).

### 3.5. Subgroup Analysis by Diagnosis

The prevalence of Class 2 membership varied substantially across diagnostic subgroups (Supplemental Figure S4A). Hemorrhagic diagnoses demonstrated the highest Class 2 proportions: SAH (19.1%, 27/141), ICH (15.0%, 15/100), and TBI (11.6%, 16/138). In contrast, elective neurosurgical conditions had much lower Class 2 proportions: unruptured aneurysm (1.0%, 14/1461) and moyamoya disease (1.0%, 3/290). Brain tumors, the largest diagnostic group, had a Class 2 proportion of 3.9% (162/4165). Among Class 2 patients, unfavorable neurological outcome rates were highest in ICH (86.7%) and TBI (62.5%), followed by SAH (51.9%) and other diagnoses (37.7%) (Supplemental Figure S4B). The association between Class 2 and unfavorable neurological outcome was statistically significant in most diagnostic subgroups (Supplemental Figure S4C): unruptured aneurysm (OR 24.4, 95% CI 6.2–95.8; *p* < 0.001), other diagnoses (OR 20.7, 95% CI 13.1–32.7; *p* < 0.001), brain tumor (OR 19.9, 95% CI 13.2–30.0; *p* < 0.001), ICH (OR 14.8, 95% CI 3.1–70.1; *p* < 0.001), and SAH (OR 4.3, 95% CI 1.8–10.3; *p* = 0.001). The association was not significant for TBI (OR 2.8, 95% CI 0.9–8.1; *p* = 0.102), likely due to the high baseline rate of unfavorable outcomes in TBI patients regardless of trajectory class. A heatmap of unfavorable outcome rates by trajectory class and diagnosis (Supplemental Figure S4D) revealed that ICH patients in Class 2 had the highest rate (86.7%), and hemorrhagic diagnoses generally showed higher unfavorable outcome rates across all trajectory classes. Because the Rapid Dropper phenotype comprised only 4.1% of the cohort, several diagnosis-specific estimates were derived from small strata and are reported with their confidence intervals and event counts; estimates such as that for unruptured aneurysm (OR 24.4; 95% CI, 6.2–95.8; *n* = 14 Class 2 patients) carry wide confidence intervals and should be interpreted as exploratory.

### 3.6. Early Prediction of Unfavorable Neurological Outcome

The predictive performance of hemoglobin trajectory features was evaluated using ROC analysis (Table 2, Supplemental Figure S2). Among individual features, hemoglobin variability (AUC 0.961) and ΔHb (AUC 0.895) showed the highest discrimination for identifying Class 2 membership, suggesting that these readily computable metrics may serve as practical surrogates for trajectory classification without requiring formal trajectory clustering (Supplemental Figure S2A). For predicting unfavorable neurological outcome, the combined model incorporating ΔHb, slope, and variability achieved an AUC of 0.850 (95% CI, 0.829–0.870) (Supplemental Figure S2B). Notably, the addition of Class 2 membership to these continuous features did not improve discrimination (AUC 0.850; cross-validated AUC 0.848), indicating that trajectory class information is fully captured by the continuous hemoglobin features alone (Supplemental Figure S2C). The combined model also demonstrated strong performance for mortality prediction, with AUCs of 0.890 (95% CI, 0.852–0.923) for ICU mortality, 0.883 (95% CI, 0.841–0.919) for in-hospital mortality, and 0.828 (95% CI, 0.787–0.867) for 28-day mortality.

Because ΔHb and variability define the phenotype, the high in-sample AUC of 0.850 for identifying Class 2 is partly tautological; we therefore present the phenotyping as descriptive of prognostically distinct groups rather than as a predictive tool. To estimate leakage-free early-prediction performance, we performed a 72 h landmark analysis: the early-feature model achieved an AUC of 0.718 (cross-validated 0.714), and ΔHb plus slope alone an AUC of 0.646 (cross-validated 0.645). Thus the full-trajectory AUC of 0.850 reflects retrospective phenotyping, whereas true early-prediction performance is approximately AUC 0.72.

When used as a binary classifier, Class 2 membership demonstrated high specificity (97.3%) and NPV (96.9%) for unfavorable neurological outcome, with sensitivity of 33.8% and PPV of 36.5% (Supplemental Figure S2F). This pattern indicates that while Class 2 identifies a high-risk subgroup with approximately one-third probability of poor outcome, many patients with unfavorable outcomes follow other trajectory patterns. The sensitivity/specificity trade-off across ΔHb cutoffs (Supplemental Figure S2D) suggests that a ΔHb threshold of 2.0–2.5 g/dL provides a reasonable balance for clinical screening purposes.

## 4. Discussion

In this large retrospective cohort study of 8517 patients admitted to a neurosurgical ICU over a decade, we identified six distinct hemoglobin trajectory phenotypes using Gaussian mixture modeling of trajectory-derived features. The principal finding of this study is that the Class 2 “Rapid Dropper” phenotype, comprising a small minority of the total cohort, exhibited a disproportionately high rate of unfavorable neurological outcomes compared with the reference class. Critically, this association remained statistically significant after rigorous propensity score matching, supporting a significant association of trajectory classification with outcome beyond measured baseline severity, although residual confounding cannot be fully excluded. Furthermore, trajectory-derived features—including hemoglobin slope, variability, and delta hemoglobin—demonstrated strong discriminative performance for predicting unfavorable outcomes. These results support our a priori hypothesis that dynamic hemoglobin trajectory patterns are significantly associated with neurological outcomes beyond static threshold measurements and are consistent with a shift from threshold-based to trajectory-based hemoglobin assessment in neurocritical care.

The prevailing clinical approach to hemoglobin management in neurocritical care has centered on identifying an optimal transfusion threshold—a binary question of whether hemoglobin should be maintained above 7–8 g/dL (restrictive) or 9–10 g/dL (liberal) [6,7,8,10]. The HEMOTION trial demonstrated no significant neurological benefit of a liberal strategy in traumatic brain injury [6], the SAH-RCT similarly showed no improvement in functional outcomes with higher hemoglobin targets in subarachnoid hemorrhage [7], and the TRAIN multicenter trial reported comparable outcomes between restrictive and liberal strategies across acute brain injury subtypes [8]. A recent systematic review and meta-analysis of randomized controlled trials further confirmed no significant differences in mortality or unfavorable outcomes between the two strategies [26]. While these findings have informed current practice, they collectively reveal a fundamental limitation of the threshold-based paradigm: it treats hemoglobin as a static variable captured at discrete time points, thereby failing to account for the dynamic nature of hemoglobin changes throughout the ICU course. Our findings directly address this gap by demonstrating that the pattern and velocity of hemoglobin decline, rather than any single absolute value, serve as a more powerful predictor of neurological outcomes. This observation aligns with the growing recognition that longitudinal biomarker trajectories outperform single measurements in prognostication across critical care populations, as demonstrated for intracranial pressure trajectories [11], albumin trajectories [18], and SOFA score trajectories [19] in ICU settings.

The pathophysiological basis for the adverse outcomes associated with rapid hemoglobin decline warrants careful consideration. Cerebral oxygen delivery is determined by the product of cerebral blood flow and arterial oxygen content, with the latter being primarily dependent on hemoglobin concentration [2,5]. In the normal brain, progressive anemia triggers compensatory cerebral vasodilation, maintaining adequate cerebral oxygen delivery until hemoglobin falls below approximately 5–6 g/dL [2,14]. However, in the injured brain, where cerebral autoregulation is frequently impaired, this compensatory mechanism is significantly attenuated, with critical thresholds of cerebral oxygen delivery occurring at higher hemoglobin levels of approximately 8–9 g/dL [3,14]. Oddo et al. demonstrated that hemoglobin concentrations below 9 g/dL were independently associated with brain tissue hypoxia and cerebral metabolic crisis in patients with poor-grade subarachnoid hemorrhage [3]. Crucially, it is not merely the absolute hemoglobin nadir that determines the degree of cerebral injury, but the rate at which oxygen-carrying capacity declines. A rapid decrease in hemoglobin, as exemplified by the Class 2 trajectory, may overwhelm the already limited cerebrovascular reserve before compensatory mechanisms can be engaged, resulting in sustained periods of cerebral hypoperfusion and metabolic distress [5,27]. Furthermore, the high hemoglobin variability observed in Class 2 patients implies repeated fluctuations in cerebral oxygen delivery, imposing cyclical perfusion challenges that compound cumulative ischemic injury to vulnerable brain tissue [4,15].

The substantial attenuation of the odds ratio after propensity score matching merits nuanced interpretation. This reduction indicates that a considerable proportion of the crude association between the Rapid Dropper phenotype and unfavorable outcomes is attributable to baseline severity differences—patients in Class 2 had lower initial GCS, and greater proportions of hemorrhagic diagnoses requiring surgical intervention. However, the persistence of a statistically significant and clinically meaningful odds ratio after comprehensive adjustment demonstrates that the hemoglobin trajectory itself carries independent prognostic information beyond these established risk factors. This finding has important clinical implications: it suggests that rapid hemoglobin decline is not merely a passive epiphenomenon reflecting disease severity but may actively contribute to secondary neurological injury through the mechanisms described above. Notably, in-hospital mortality and ICU mortality showed more modest effect sizes after propensity score matching, with ICU mortality reaching borderline significance. This pattern likely reflects the fact that mortality is influenced by numerous non-neurological factors, whereas the GCS captures the cumulative burden of both primary and secondary brain injury, making it a more sensitive endpoint for trajectory-mediated effects on neural tissue integrity.

Subgroup analysis by diagnosis revealed that hemorrhagic conditions were overrepresented in the Rapid Dropper phenotype: SAH, ICH, and TBI patients classified as Class 2 at substantially higher rates than those in other diagnostic categories. Those with ICH exhibited the highest rate of unfavorable neurological outcomes, markedly exceeding the rates observed in Class 2 patients with SAH or TBI. The disproportionate representation of hemorrhagic diagnoses in Class 2 is mechanistically consistent with the pathophysiology of these conditions: ongoing hemorrhage, repeated surgical blood loss, hemodilution from aggressive fluid resuscitation, and coagulopathy-related bleeding collectively contribute to precipitous hemoglobin decline [2,28]. In ICH specifically, the exceptionally poor outcomes likely reflect the convergent effects of direct parenchymal destruction, perihematomal edema, mass effect, and superimposed secondary ischemic injury from rapid hemoglobin decline [29,30]. For TBI, the association between Class 2 and unfavorable outcomes showed a trend but did not reach statistical significance in the propensity score–matched subgroup analysis, which may be attributable to the already high baseline rate of unfavorable outcomes in this population, limiting the statistical power to detect an incremental effect of hemoglobin trajectory [6]. These diagnosis-specific findings suggest that hemoglobin trajectory monitoring may be particularly valuable in patients with hemorrhagic stroke, where the risk of rapid hemoglobin decline is highest and the potential for targeted intervention is greatest [31].

A notable strength of this study is the demonstration that simple, bedside-calculable hemoglobin trajectory features achieve robust discriminative performance for predicting unfavorable neurological outcomes. The combined model incorporating delta hemoglobin, slope, and variability yielded excellent discriminative performance, rivaling or exceeding the performance of more complex prognostic scoring systems commonly used in neurocritical care [1,32]. Importantly, these trajectory-derived features do not require the computational complexity of formal trajectory clustering; delta hemoglobin, slope, and standard deviation can be readily calculated from serial hemoglobin measurements that are already routinely obtained in the ICU setting. Class 2 demonstrated high specificity for unfavorable outcomes, indicating that identification of this trajectory pattern functions effectively as a “rule-in” strategy—when a patient is classified as a Rapid Dropper, the probability of poor outcome is very high. Our data suggest that a delta hemoglobin of 2.0–2.5 g/dL within the first 48–72 h of ICU admission could serve as a practical screening cutoff, below which the risk of unfavorable outcome increases substantially. This threshold could be integrated into existing clinical workflows and electronic health record systems as an automated early warning trigger, facilitating timely clinical reassessment and potentially initiating targeted interventions before irreversible neurological injury occurs [33].

The clinical implications of our findings extend beyond prognostication and into the realm of transfusion strategy. The current debate, framed as “restrict versus liberalize,” implicitly assumes that a single hemoglobin threshold should govern transfusion decisions for all neurocritical patients at all time points. Our data challenges this assumption by demonstrating that the velocity of hemoglobin change is a critical determinant of outcome, independent of absolute hemoglobin level. We tentatively propose, as a hypothesis requiring prospective validation, the concept of a “velocity-based transfusion trigger,” in which the rate of hemoglobin decline over the first 48–72 h could serve as a complementary criterion for transfusion decision-making [20]. In practical terms, this approach would involve systematic monitoring of hemoglobin trends from serial measurements, with calculation of the hemoglobin slope (change per day) and delta hemoglobin at predefined intervals. Patients exhibiting a hemoglobin decline exceeding a critical velocity (e.g., >0.10 g/dL/day) or a cumulative delta hemoglobin exceeding 2.0 g/dL within 48–72 h would be flagged for clinical reassessment, regardless of whether their absolute hemoglobin has crossed a conventional threshold. This approach would enable proactive identification of high-risk patients and allow clinicians to consider early interventions—including investigation and management of the underlying cause of hemoglobin decline, optimization of volume status, and consideration of transfusion—before the hemoglobin decline progresses further. Such a strategy does not replace threshold-based guidelines but augments them with a dynamic, patient-specific layer of risk assessment that captures the temporal dimension of hemoglobin management [13,34,35].

This study has several limitations that warrant consideration. First, as a single-center retrospective study conducted at a tertiary neurosurgical ICU, our findings may not be directly generalizable to other institutional settings with different patient populations, transfusion practices, or clinical protocols. The predominance of patients from a single geographic and ethnic background may also limit external validity. Second, the retrospective design precludes establishing a causal relationship between hemoglobin trajectory and neurological outcomes; the observed association may be influenced by reverse causality, as transfusion decisions themselves alter the hemoglobin trajectory and may reflect clinical judgment about disease severity [2]. Third, the GOS was assessed at hospital discharge rather than at a standardized long-term follow-up time point (e.g., 6 or 12 months), which may not fully capture the final neurological recovery of patients with acute brain injury, particularly those with traumatic brain injury who may show delayed improvement [6,9]. Fourth, the Gaussian mixture modeling approach, while statistically grounded, involves a degree of subjectivity in selecting the optimal number of phenotypes, and different feature specifications or interpolation choices could yield alternative groupings; accordingly, the precise boundaries of the six-phenotype solution should be regarded as exploratory, whereas the prognostic signal of the observed hemoglobin decline was robust regardless of these specifications; in a no-interpolation sensitivity analysis the exact six-way partition was sensitive to interpolation (adjusted Rand index ≈ 0.14 vs. the reported labels), yet using observed values only the prognostic signal was preserved (observed day-to-day decline +2.2 vs. 0.0 g/dL; observed nadir 8.9 vs. 10.6 g/dL; observed ΔHb AUC 0.70; observed nadir AUC 0.77; 12.2% vs. 5.6% unfavorable GOS in the steepest observed-decline decile) [16,17]. We addressed this by employing established model selection criteria including the Bayesian information criterion, average posterior probability, and entropy. Fifth, we did not have data on transfusion timing, storage age of red blood cells, or detailed cerebral monitoring parameters such as brain tissue oxygenation, which could provide additional mechanistic insights into the relationship between hemoglobin trajectory and cerebral physiology [3,27]. Finally, the relatively low prevalence of Class 2 (4.1%) limits the statistical power for subgroup analyses, particularly within individual diagnostic categories.

Several additional limitations follow from the revised analyses. First, although the association of the Rapid Dropper phenotype with unfavorable outcome persisted after adjustment for illness severity (APACHE II) and treatment intensity, residual confounding cannot be excluded, and we therefore describe these findings as significant associations rather than as independent or causal effects. Second, the full-trajectory model is retrospective by construction: because ΔHb and variability define the phenotype, its high in-sample AUC (0.850) is partly tautological, and the clinically meaningful, leakage-free estimate is the 72 h early-prediction AUC of approximately 0.72; the landmark analysis may also be affected by survivorship and ascertainment bias. Third, the primary outcome—discharge GOS—depends on local discharge practices and does not capture long-term neurological recovery, a limitation compounded by the heterogeneous case mix, so that our interpretation is restricted to short-term functional status. Fourth, because Class 2 comprised only 4.1% of the cohort, the robust associations observed in the overall and never-transfused analyses (adjusted OR for unfavorable GOS 4.98 and 6.56, respectively) should be distinguished from the diagnosis-specific subgroup estimates, whose small strata and wide confidence intervals render the precise six-phenotype boundaries exploratory. Finally, estimated surgical blood loss was not recorded and could not be adjusted for, and a perioperative baseline hemoglobin was available in only 11.5% of patients, so that the perioperative decline analysis is exploratory.

## 5. Conclusions

In conclusion, this study demonstrates that hemoglobin trajectory phenotyping using Gaussian mixture modeling of hemoglobin trajectory features provides a novel dimension for risk stratification in this neurosurgical ICU cohort. The identification of a “Rapid Dropper” phenotype—characterized by steep hemoglobin decline, high variability, and significantly associated with 2.4-fold higher odds of unfavorable neurological outcome—offers clinicians a previously unrecognized prognostic marker. The clinical accessibility of trajectory-derived features (delta hemoglobin, slope, variability) enables real-time risk assessment at the bedside without the need for complex computational tools. These associations require prospective and external validation before they can inform transfusion strategy. Future prospective, multicenter studies are warranted to validate these trajectory phenotypes, explore candidate velocity-based thresholds, and ultimately evaluate whether trajectory-guided transfusion strategies can improve neurological outcomes in this vulnerable patient population.

## Figures and Tables

**Table 1:** Baseline Characteristics and Trajectory Features by Hemoglobin Trajectory Phenotype.

Variable	Total (*n* = 8517)	Class 1 (*n* = 922)	Class 2 (*n* = 351)	Class 3 (*n* = 2738)	Class 4 (*n* = 751)	Class 5 (*n* = 2611)	Class 6 (*n* = 1144)	*p*
Demographics								
Age, years	55.2 ± 14.5	54.3 ± 14.8	54.7 ± 16.7	56.3 ± 13.1	52.8 ± 16.6	55.5 ± 14.1	54.2 ± 15.5	<0.001
Male sex	3573 (41.9)	411 (44.6)	206 (58.7)	1005 (36.7)	289 (38.5)	1043 (39.9)	619 (54.1)	<0.001
GCS at admission	14.4 ± 1.8	14.5 ± 1.7	11.7 ± 4.3	14.9 ± 0.8	13.6 ± 2.8	14.7 ± 1.0	14.2 ± 2.3	<0.001
Primary Diagnosis								
SAH	141 (1.7)	11 (1.2)	27 (7.7)	14 (0.5)	21 (2.8)	37 (1.4)	31 (2.7)	
ICH	100 (1.2)	16 (1.7)	15 (4.3)	10 (0.4)	15 (2.0)	19 (0.7)	25 (2.2)	
TBI	138 (1.6)	10 (1.1)	16 (4.6)	18 (0.7)	39 (5.2)	26 (1.0)	29 (2.5)	
Brain tumor	4165 (48.9)	505 (54.8)	162 (46.2)	1019 (37.2)	453 (60.3)	1510 (57.8)	516 (45.1)	
Unruptured aneurysm	1461 (17.2)	114 (12.4)	14 (4.0)	865 (31.6)	25 (3.3)	267 (10.2)	176 (15.4)	
Moyamoya disease	290 (3.4)	31 (3.4)	3 (0.9)	113 (4.1)	12 (1.6)	88 (3.4)	43 (3.8)	
Other	2222 (26.1)	235 (25.5)	114 (32.5)	699 (25.5)	186 (24.8)	664 (25.4)	324 (28.3)	<0.001 ^†^
Comorbidities								
Hypertension	844 (9.9)	102 (11.1)	61 (17.4)	243 (8.9)	74 (9.9)	242 (9.3)	122 (10.7)	<0.001
Diabetes mellitus	509 (6.0)	57 (6.2)	36 (10.3)	120 (4.4)	47 (6.3)	156 (6.0)	93 (8.1)	<0.001
Malignancy	5260 (61.8)	637 (69.1)	246 (70.1)	1303 (47.6)	567 (75.5)	1795 (68.7)	712 (62.2)	<0.001
Cardiovascular disease	69 (0.8)	8 (0.9)	8 (2.3)	15 (0.5)	7 (0.9)	15 (0.6)	16 (1.4)	0.002
Cerebrovascular disease	2721 (31.9)	237 (25.7)	108 (30.8)	1191 (43.5)	157 (20.9)	637 (24.4)	391 (34.2)	<0.001
Hemoglobin Parameters								
First Hb, g/dL	11.9 ± 1.7	12.1 ± 1.8	12.6 ± 2.1	12.0 ± 1.4	11.2 ± 1.8	11.6 ± 1.6	12.7 ± 1.7	<0.001
Minimum Hb, g/dL	10.7 ± 1.7	10.7 ± 1.7	8.9 ± 1.6	11.2 ± 1.3	9.1 ± 1.5	10.9 ± 1.5	10.6 ± 1.7	<0.001
Maximum Hb, g/dL	12.5 ± 1.5	12.5 ± 1.6	13.3 ± 1.6	12.2 ± 1.3	12.5 ± 1.5	12.4 ± 1.5	13.0 ± 1.6	<0.001
Delta Hb, g/dL	1.3 ± 1.2	1.3 ± 0.9	3.6 ± 1.7	0.8 ± 0.7	2.0 ± 1.5	0.7 ± 0.8	2.0 ± 1.0	<0.001
Hb slope, g/dL/day	0.006 ± 0.08	0.012 ± 0.02	–0.135 ± 0.12	–0.031 ± 0.02	0.132 ± 0.08	0.066 ± 0.04	–0.079 ± 0.04	<0.001
Hb variability (SD)	0.45 ± 0.29	0.36 ± 0.11	1.09 ± 0.34	0.25 ± 0.12	0.90 ± 0.27	0.40 ± 0.20	0.58 ± 0.14	<0.001
Hb measurement count	5.2 ± 3.1	5.4 ± 2.5	12.4 ± 5.0	3.7 ± 1.2	8.0 ± 3.9	4.6 ± 1.7	6.1 ± 3.8	<0.001
Interventions								
Mechanical ventilation	1191 (14.0)	133 (14.4)	205 (58.4)	152 (5.6)	234 (31.2)	243 (9.3)	224 (19.6)	<0.001
ICP monitoring	640 (7.5)	76 (8.2)	133 (37.9)	64 (2.3)	114 (15.2)	106 (4.1)	147 (12.8)	<0.001
CRRT	22 (0.3)	0 (0)	13 (3.7)	2 (0.1)	4 (0.5)	1 (0.0)	2 (0.2)	<0.001
Transfusion								
RBC transfusion	1475 (17.3)	168 (18.2)	162 (46.2)	215 (7.9)	339 (45.1)	396 (15.2)	195 (17.0)	<0.001
RBC units	0.9 ± 4.5	0.7 ± 2.7	5.4 ± 14.6	0.2 ± 1.1	3.1 ± 7.7	0.5 ± 1.9	0.9 ± 4.6	<0.001

Values are presented as mean ± SD or *n* (%). *p* values were calculated using the Kruskal–Wallis test for continuous variables and the chi-squared test for categorical variables. ^†^ *p* value for overall comparison across all diagnosis categories. Abbreviations: SAH, subarachnoid hemorrhage; ICH, intracerebral hemorrhage; TBI, traumatic brain injury; GCS, Glasgow Coma Scale; Hb, hemoglobin; ICP, intracranial pressure; CRRT, continuous renal replacement therapy; RBC, red blood cell.

**Table 2:** Predictive Performance of Hemoglobin Trajectory Features for Clinical Outcomes.

Predictor/Model	AUC	95% CI	Sensitivity (%)	Specificity (%)	CV-AUC
Predicting Class 2 membership					
Delta Hb	0.895	0.875–0.914	–	–	–
Slope	0.868	0.848–0.886	–	–	–
Variability (Hb SD)	0.961	0.955–0.966	–	–	–
Predicting unfavorable GOS (individual features)					
Delta Hb	0.811	0.785–0.837	–	–	–
Slope	0.670	0.634–0.704	–	–	–
Variability (Hb SD)	0.810	0.789–0.832	–	–	–
Minimum Hb	0.790	0.765–0.817	–	–	–
Class 2 status (binary)	0.655	0.633–0.678	33.8	97.3	–
Combined models					
Hb features *	0.850	0.829–0.870	–	–	–
Hb features + Class 2 *	0.850	0.830–0.871	–	–	0.848
Combined model + Class 2 by outcome *					
Unfavorable GOS	0.850	0.830–0.871	33.8	97.3	0.848
In-hospital mortality	0.883	0.842–0.919	35.6	96.2	0.874
28-day mortality	0.828	0.788–0.866	33.6	96.4	0.823
ICU mortality	0.890	0.853–0.923	36.5	96.2	0.884

* Combined hemoglobin features include ΔHb, slope, and variability (SD). Sensitivity and specificity for Class 2 status reflect Class 2 membership as a binary classifier. 95% confidence intervals were derived from 1000 bootstrap resamples. CV-AUC denotes 5-fold stratified cross-validated AUC. Abbreviations: AUC, area under the receiver operating characteristic curve; CI, confidence interval; CV, cross-validation; GOS, Glasgow Outcome Scale; Hb, hemoglobin.

## Data Availability

The data presented in this study are available on reasonable request from the corresponding author. The data are not publicly available because they consist of de-identified clinical records subject to institutional and patient-privacy restrictions.

## Supplementary Materials

The following supporting information can be downloaded at: https://www.mdpi.com/article/10.3390/jcm15135254/s1. Table S1: Candidate model comparison for the Gaussian mixture model (full covariance; N = 8517); Table S2: Posterior classification probabilities and sizes of the six hemoglobin trajectory phenotypes; Table S3: Sensitivity analyses—adjusted odds ratios for the Rapid Dropper phenotype (Class 2) across alternative models; Table S4: Intensive care unit and hospital length of stay by hemoglobin trajectory phenotype (full cohort); Figure S1: Study Flow Diagram; Figure S2: Prediction of Class 2 Membership and Clinical Outcome Performance; Figure S3: Propensity Score Matching: Class 2 vs. Others (1:2); Figure S4: Subgroup Analysis by Primary Neurosurgical Diagnosis.
