# Supplementary material for: Hemoglobin Trajectory Phenotypes and Neurological Outcomes in a Neurosurgical ICU Cohort
Source: J Clin Med. 2026 Jul 5;15(13):5254. doi: 10.3390/jcm15135254 (PMC13363624; doi:10.3390/jcm15135254)
Supplement: Supplementary file 1 [file jcm-15-05254-s001.zip › jcm-4397478-supplementary.pdf]

Additional file S1: Supplementary figures

Figure S1. Study Flow Diagram. Flow diagram illustrating patient selection from the initial cohort of 13,663 patients admitted to the neurosurgical ICU between January 2015 and December 2024 at Samsung Medical Center. After sequential exclusion for age < 18 years (n = 423), DNR orders (n = 689), transfer from other hospitals with insufficient data (n = 1,300), incomplete medical records (n = 872), incomplete hemoglobin data (n = 774), missing GOS data (n = 821), and non-neurocritical diagnosis (n = 267), the final study cohort comprised 8,517 patients. A feature-based Gaussian mixture model identified a six-phenotype solution as optimal, yielding: Class 1, Stable Intermediate (n = 922, 10.8%); Class 2, Rapid Dropper (n = 351, 4.1%); Class 3, Stable Maintainer (n = 2,738, 32.1%); Class 4, Low Start Recovery (n = 751, 8.8%); Class 5, Stable Mild Rise (n = 2,611, 30.7%); and Class 6, Gradual Decliner (n = 1,144, 13.4%).

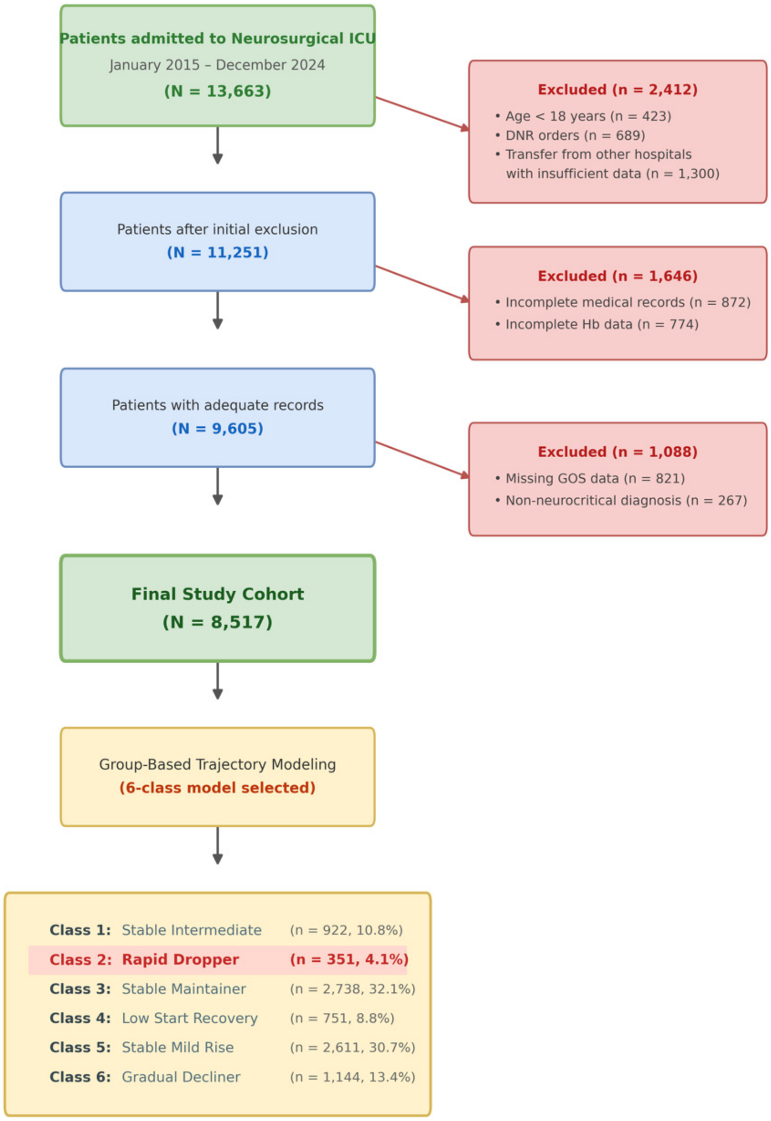

Figure S2. Prediction of Class 2 Membership and Clinical Outcome Performance. (A) ROC curves for individual hemoglobin features predicting Class 2 membership. Variability (AUC = 0.961), delta Hb (AUC = 0.895), and slope (AUC = 0.868) all demonstrated strong discrimination. (B) ROC curves for the combined hemoglobin feature model (ΔHb, slope, and variability) predicting each clinical outcome: ICU mortality (AUC = 0.890), in-hospital mortality (AUC = 0.883), unfavorable GOS (AUC = 0.850), and 28-day mortality (AUC = 0.828). (C) ROC curves for the combined hemoglobin features plus Class 2 status model, with both apparent AUC and 5-fold cross-validated AUC (CV) reported. The addition of Class 2 status provided marginal improvement, suggesting that trajectory class information is largely captured by the continuous hemoglobin features. (D) Sensitivity and specificity trade-off for Class 2 classification across delta Hb cutoffs. Reference lines indicate cutoffs of 1.5, 2.0, and 2.5 g/dL. (E) Distribution of delta Hb values in Class 2 (n = 351) versus other classes (n = 8,166), with reference lines at cutoffs of 1.5 and 2.0 g/dL. (F) Classification performance of Class 2 membership as a binary predictor: unfavorable GOS (sensitivity 33.8%, specificity 97.3%, PPV 36.5%, NPV 96.9%); in-hospital mortality (sensitivity 35.6%, specificity 96.2%, PPV 8.8%, NPV 99.3%); 28-day mortality (sensitivity 33.6%, specificity 96.4%, PPV 13.4%, NPV 98.9%); ICU mortality (sensitivity 36.5%, specificity 96.2%, PPV 8.8%, NPV 99.3%).

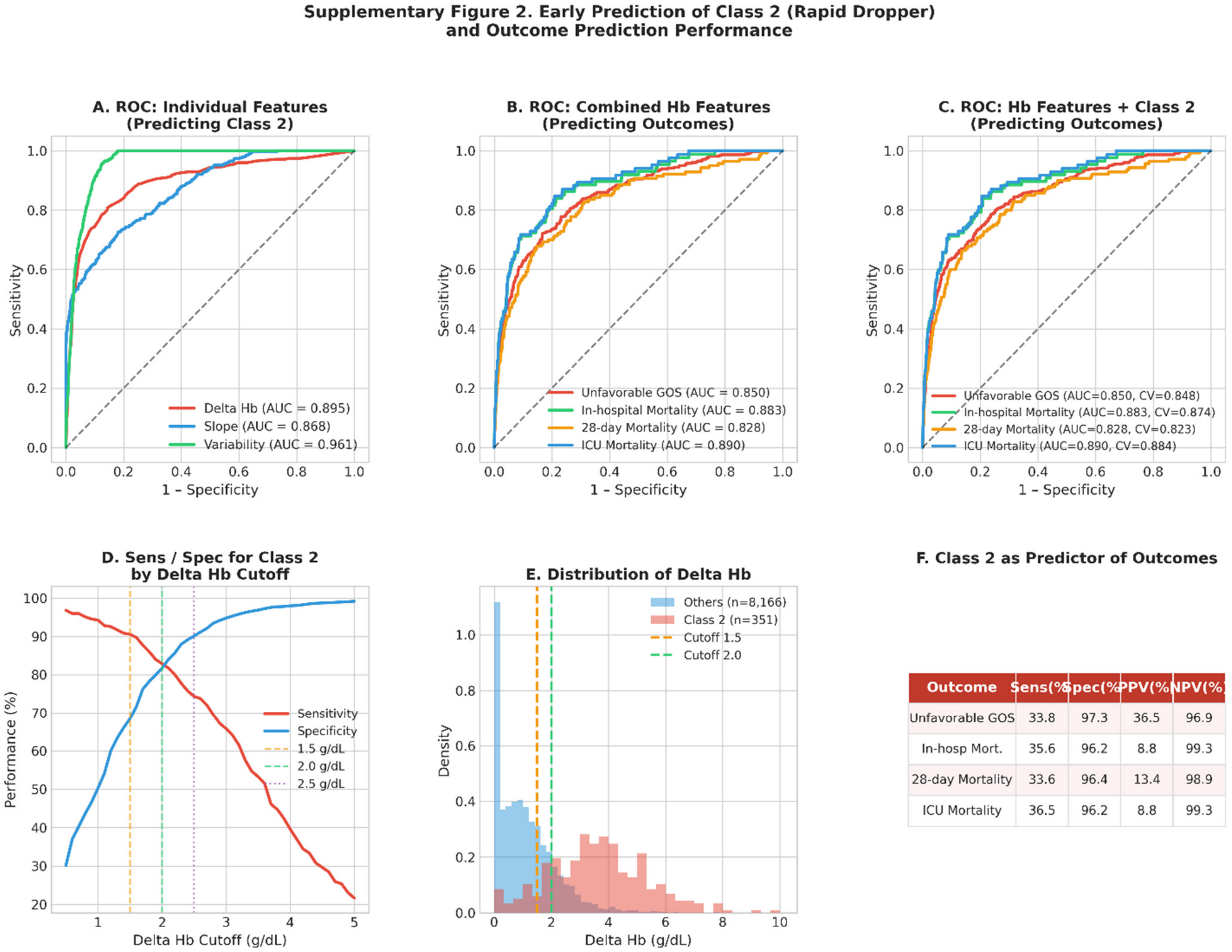

Figure S3. Propensity Score Matching: Class 2 vs. Others (1:2). (A) Propensity score distributions before matching in Class 2 (n = 351) and other classes (n = 8,166). Class 2 patients showed a wider and more right-skewed distribution, reflecting their distinct clinical profile. (B) Love plot showing standardized mean differences (SMDs) before (red circles) and after (blue squares) propensity score matching. All covariates achieved adequate balance after matching (SMD < ±0.1, green shaded zone), including age, sex, initial hemoglobin, GCS, comorbidities, and diagnostic categories. (C) Comparison of unadjusted (before) versus matched (after) odds ratios for all outcomes. Unadjusted ORs ranged from 13.4 to 18.1, substantially attenuated after PSM to 1.6–2.4, indicating that baseline severity differences contributed to but did not fully explain the observed associations. (D) Forest plot of adjusted odds ratios after 1:2 propensity score matching (329 Class 2 patients vs. 643 matched controls). Unfavorable GOS (OR 2.40, 95% CI 1.77–3.26; P < 0.001) and 28-day mortality (OR 2.09, 95% CI 1.33–3.28; P = 0.002) remained statistically significant. In-hospital mortality (OR 1.59, 95% CI 0.93–2.70; P = 0.090) and ICU mortality (OR 1.64, 95% CI 0.96–2.80; P = 0.085) showed trends toward significance.

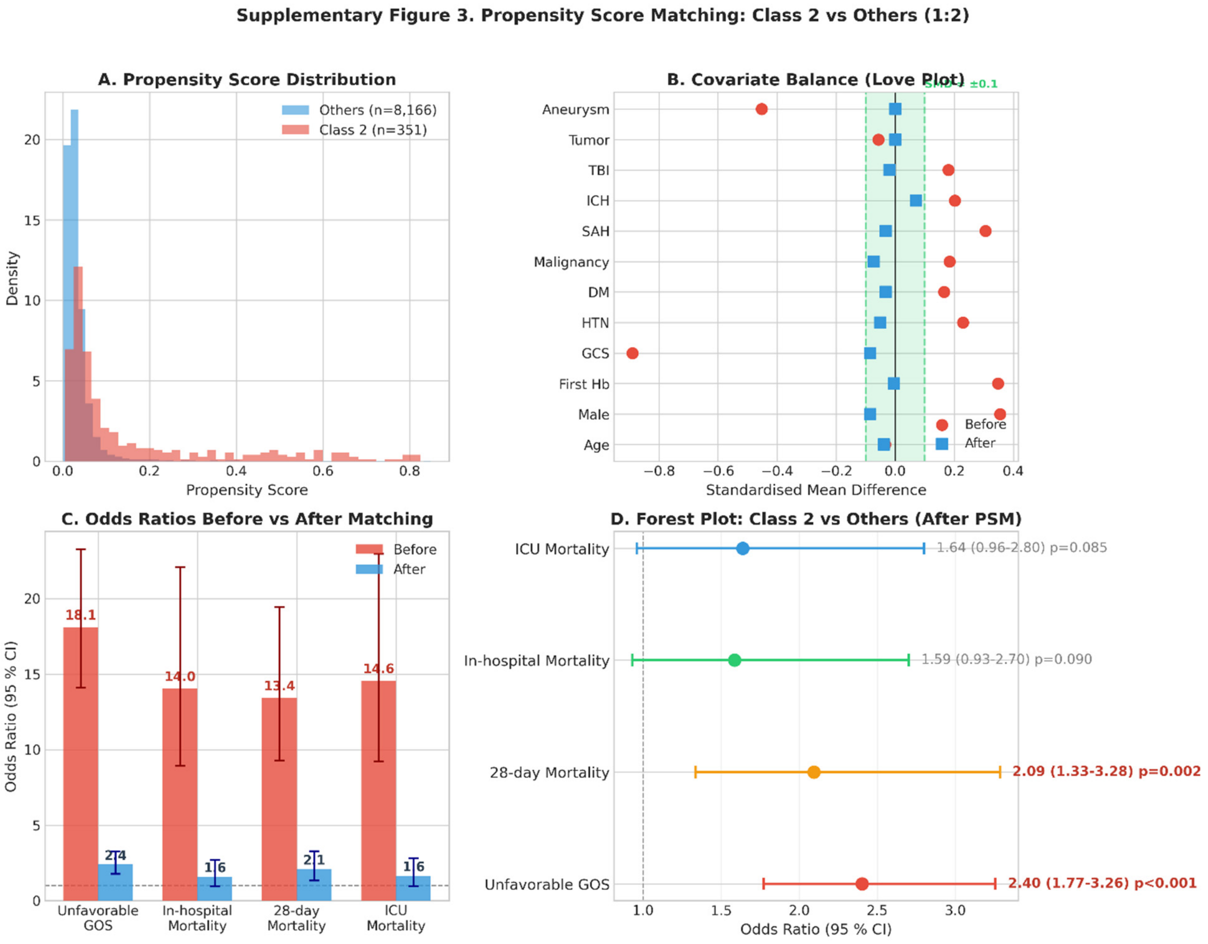

Figure S4. Subgroup Analysis by Primary Neurosurgical Diagnosis. (A) Proportion of Class 2 membership by diagnosis. Hemorrhagic diagnoses had the highest Class 2 proportions: SAH (19.1%, 27/141), ICH (15.0%, 15/100), and TBI (11.6%, 16/138). Elective conditions showed lower proportions: brain tumor (3.9%, 162/4,165) and unruptured aneurysm (1.0%, 14/1,461). The overall Class 2 proportion was 4.1% (dashed line). (B) Unfavorable neurological outcome rates among Class 2 patients by diagnosis. ICH (86.7%), TBI (62.5%), and SAH (51.9%) had the highest rates. (C) Forest plot of odds ratios (95% CI) for unfavorable GOS comparing Class 2 versus other classes within each diagnostic subgroup. Statistically significant associations were observed for unruptured aneurysm (OR 24.4, 95% CI 6.2–95.8; P < 0.001), other diagnoses (OR 20.7, 95% CI 13.1–32.7; P < 0.001), brain tumor (OR 19.9, 95% CI 13.2–30.0; P < 0.001), ICH (OR 14.8, 95% CI 3.1–70.1; P < 0.001), and SAH (OR 4.3, 95% CI 1.8–10.3; P = 0.001). TBI showed a non-significant trend (OR 2.8, 95% CI 0.9–8.1; P = 0.102). (D) Heatmap of unfavorable outcome rates (%) by trajectory class and diagnosis. ICH patients in Class 2 exhibited the highest rate (87%), and hemorrhagic diagnoses generally showed elevated rates across multiple trajectory classes.

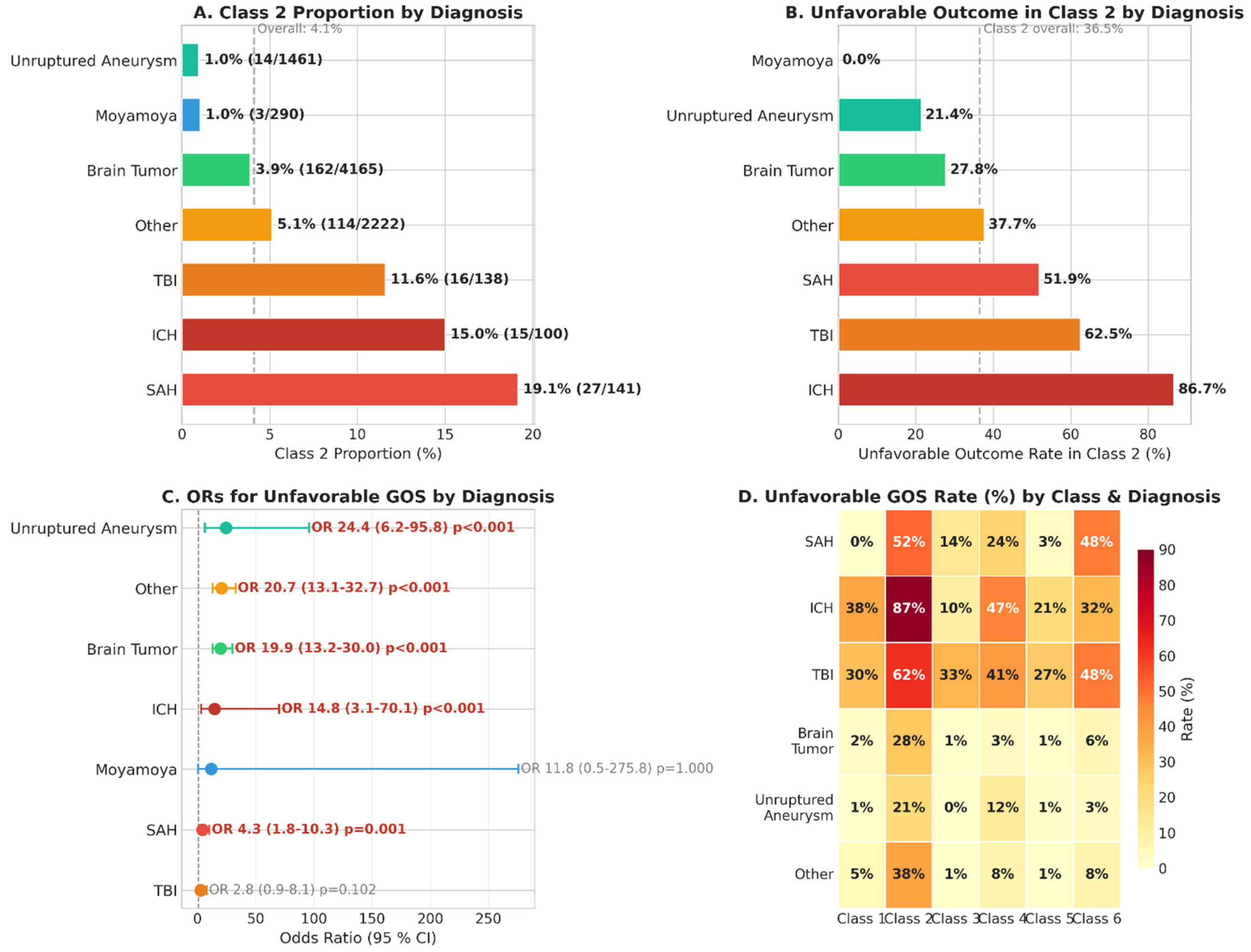

Supplementary Table S1. Candidate model comparison for the Gaussian mixture model (full covariance; N = 8,517).

K	Log-likelihood	No. of parameters	AIC	BIC	Relative entropy	Smallest class (%)
2	−31,205.5	19	62,449.0	62,583.0	0.612	41.3
3	−28,783.2	29	57,624.5	57,828.9	0.746	12.8
4	−28,276.3	39	56,630.6	56,905.6	0.704	7.4
5	−27,656.8	49	55,411.6	55,757.1	0.708	8.6
**6**	**−27,356.9**	**59**	**54,831.7**	**55,247.6**	**0.686**	**5.7**
7	−27,221.2	69	54,580.3	55,066.8	0.661	5.2
8	−27,073.5	79	54,305.0	54,861.9	0.680	2.4

AIC, Akaike information criterion; BIC, Bayesian information criterion. Phenotyping was performed over a pre-specified range of two to six components; the six-component solution (bold) was selected. Models with seven to eight components are shown for completeness. The incremental BIC improvement diminishes substantially beyond six components (successive ΔBIC 1,149 → 509 → 181 → 205), relative entropy does not improve, and additional components become clinically uninterpretably small (smallest class 2.4% at K = 8). The six-component solution was retained as the balance of fit, parsimony, and interpretability.

Supplementary Table S2. Posterior classification probabilities and sizes of the six hemoglobin trajectory phenotypes.

Trajectory phenotype	n	%	Average posterior probability (APPA)
Class 1 — Stable Intermediate	922	10.8	0.673
**Class 2 — Rapid Dropper**	**351**	**4.1**	**0.958**
Class 3 — Stable Maintainer	2,738	32.1	0.718
Class 4 — Low Start Recovery	751	8.8	0.850
Class 5 — Stable Mild Rise	2,611	30.7	0.771
Class 6 — Gradual Decliner	1,144	13.4	0.788

APPA, average posterior probability of assignment. All phenotypes showed APPA ≥ 0.67, indicating adequate classification certainty; the high-risk Rapid Dropper phenotype (bold) was the most cleanly separated (0.958).

Supplementary Table S3. Sensitivity analyses: adjusted odds ratios for the Rapid Dropper phenotype (Class 2) across alternative models.

Outcome	Base model (12 covariates)	+ APACHE II & treatment intensity	+ Transfusion-adjusted	Never-transfused subgroup	+ Hb measurement count
Unfavorable GOS	7.73 (5.23–11.43)	4.98 (3.31–7.50)	6.67 (4.50–9.90)	6.56 (3.57–12.04)	2.71 (1.74–4.20)
28-day mortality	2.88 (1.75–4.74)	2.49 (1.52–4.08)	2.61 (1.59–4.30)	3.44 (1.53–7.72)	3.08 (1.79–5.31)
ICU mortality	1.64 (0.87–3.09)	1.57 (0.84–2.94)	—	—	—
In-hospital mortality	1.54 (0.81–2.90)	1.52 (0.81–2.85)	—	—	—

OR, odds ratio; CI, confidence interval; APACHE II, Acute Physiology and Chronic Health Evaluation II; CRRT, continuous renal replacement therapy; GOS, Glasgow Outcome Scale; Hb, hemoglobin. Values are adjusted odds ratios (95% CI) for the Rapid Dropper phenotype versus all other phenotypes, all $p < 0.05$ unless otherwise noted. The base model adjusts for the 12 pre-specified covariates (age, sex, initial hemoglobin, GCS, hypertension, diabetes mellitus, malignancy, and primary diagnosis); the propensity-score–matched estimate reported in the main text is more conservative. Treatment intensity comprises mechanical ventilation, intracranial pressure monitoring, and CRRT. The never-transfused subgroup comprised 7,042 patients (Class 2, n = 189); transfusion-adjusted and measurement-count models used the full cohort. Em dash (—), not assessed.

Supplementary Table S4. Intensive care unit and hospital length of stay by hemoglobin trajectory phenotype (full cohort).

Trajectory phenotype	n	ICU LOS, days	Hospital LOS, days
1 Stable, mid	922	0.9 [0.8–1.1]	10.0 [7.8–17.5]
**2 Rapid Dropper**	**351**	**3.1 [0.9–11.6]**	**27.8 [15.2–51.1]**
3 Stable, low variability	2,738	1.0 [0.8–1.0]	7.8 [5.8–9.8]
4 Low start, recovery	751	1.0 [0.8–2.8]	16.7 [10.8–31.8]
5 Mild rise	2,611	0.9 [0.8–1.0]	8.9 [7.7–12.8]
6 High, declining	1,144	1.0 [0.8–1.6]	10.7 [6.9–19.8]
**Overall**	**8,517**	**1.0 [0.8–1.1]**	**8.9 [6.9–14.7]**

IQR, interquartile range; ICU, intensive care unit; LOS, length of stay. Values are median [IQR] days for the full cohort. The Rapid Dropper phenotype (Class 2) had markedly longer ICU and hospital stays than all other phenotypes (Kruskal–Wallis $p < 0.001$ for both; Class 2 vs. others $p < 0.001$).
